
JOURNAL INFORMATION
==============================
NLM Title Abbreviation: ScientificWorldJournal
Journal ID: ScientificWorldJournal
NLM Journal ID: 101131163
Journal ID: TSWJ

The Scientific World Journal

ISSN: 2356-6140
EISSN: 1537-744X
Publisher: Wiley

ARTICLE INFORMATION
==============================
PMCID: PMC5848737
PMID: 18758664
DOI: 10.1100/tsw.2008.102
Article ID: 125489
Article version: 1
Subjects: Editorial

Cardiac Development

Poelmann Robert E. *
Gittenberger-de Groot Adriana C.
Department of Anatomy and Embryology, Leiden University Medical Center, Leiden, The Netherlands
*Robert E. Poelmann: r.e.poelmann@lumc.nl
Publication date: 2008
Electronic publication date: 2008 Aug 31
Volume: 8
First page: 855
Last page: 858
Received 2008 Jul 15; Accepted 2008 Jul 20
Copyright: Copyright © 2008 Robert E. Poelmann and Adriana C. Gittenberger-de Groot.
Copyright year: 2008
License: This is an open access article distributed under the Creative Commons Attribution License, which permits unrestricted use, distribution, and reproduction in any medium, provided the original work is properly cited.
License URL: https://creativecommons.org/licenses/by/3.0/

==============================
Editorial
Special Issue: Cardiac Development
*TheScientificWorldJOURNAL* (2008) 8, 855–858
TSW Development & Embryology
ISSN 1537-744X; DOI 10.1100/tsw.2008.102

TheScientificWorldJOURNAL
www.thescientificworld.com

### Cardiac Development

Robert E. Poelmann* and Adriana C. Gittenberger-de Groot

*Department of Anatomy and Embryology, Leiden University Medical Center, Leiden, The Netherlands*

**E-mail:** R.E.Poelmann@lumc.nl

*Received July 15, 2008; Accepted July 20, 2008; Published August 31, 2008*

---

The development of the cardiovascular system is a very intriguing issue as it concerns the first functional organ in the embryo. In nearly 50% of prenatally lethal mouse knock-outs, the cardiovascular/hemopoietic system is affected, underscoring the importance for embryo survival[1,2].

Fortunately, the heart is present in many taxons of the animal kingdom (including insects to chordates, from the fruitfly to tunicates, and mammals), providing the opportunity to study specific mechanisms in the most favorable species. It is evident that the simple, almost straight, tube hearts, both in development and evolution, finally emerge into the more complex form of a four-chambered double-circulation heart in adult birds and mammals, containing both a pulmonary and a systemic circuit. Moreover, terrestrial Anurans are provided with additional lymph hearts.

It is small wonder that the complexity of heart development often results in malformations, the largest group of congenital anomalies, at least in the western world. It has to be kept in mind, however, that a large number of conceptuses will not even reach full term, many because of an anomalous cardiovascular system. Many nonlethal cardiovascular anomalies are also encountered that nevertheless need clinical intervention to improve quality of life, providing a huge cost factor for society.

Studying heart development has not only a medical impact. Many genes and cell populations are involved in cardiac-specific mechanisms, such as cardiogenesis, looping, septation, valve formation, pump function, and hemodynamics. More generic processes, such as epithelium-to-mesenchyme transformation or EMT (in the endocardium to form the endocardial cushions, and in the epicardium to form epicardium-derived cells), cell migration (neural crest cells towards both the arterial and the venous pole), ion channel function (central and peripheral conduction system), and apoptosis (neural crest cells, myocardium) play important roles as well. As the cells' transduction machinery is composed of many interlocking pathways, the above-mentioned mechanisms usually interlock and cannot be separated easily.

This special issue on Cardiac Development of *TheScientificWorldJOURNAL* is devoted to many of these mechanisms and interactions, starting as early as the precardiac mesoderm. The paper by Eisenberg and Eisenberg[3] deals with Wnt signal transduction pathways that start in precardiac stages. The importance of canonical Wnt signaling is still not proven, while the influence of Wnt11, Dickkopf, and crescent has not been demonstrated to be inductive. Nevertheless, the distribution of Wnts, Wnt receptors, and inhibitors suggests a significant impact on heart development. As the Eisenbergs argue, a large number of events in cardiac development, including movement of the precardiac fields, heart tube formation, and EMT, are regulated by Wnt signaling.

One of the major issues in cardiac development is the change of 3-D geometry, starting with tube formation after midline fusion of the bilateral cardiogenic fields. The tube will loop into a C-shaped tube, continuing into a more twisted S shape. This process is generally referred to as looping.

*Corresponding author.
©2008 with author.
Published by TheScientificWorld; www.thescientificworld.com

The contribution by Linask and VanAuker[4] analyzes the role of the cytoskeleton in the laterality and directionality of cardiac looping. It has to be realized that looping involves the earliest part of the heart, the primary heart tube, which derives from the precardiac fields, but participation of the second heart field drives further development and addition of, e.g., the atria, the larger part of the right ventricle and the outflow tract. RhoA regulates morphogenetic events by altering extracellular matrix (ECM)/cell surface/cytoskeletal interactions at focal contacts involving mechanotransduction. Flectin is an important intermediary between ECM and myocardium. The biomechanics of heart looping depend on local differences in wall stiffness, illustrated by the softness of the cardiac cushions compared to the myocardium, while the inner curvature is stiffer than the outer curvature. Furthermore, material properties change with increasing intracardiac pressure, e.g., as result of chamber septation.

Cardiac development depends not only on the cardiac lineages *per se*, but also on extracardiac contributions. This is paramount for the neural crest[5]. In early embryos, the cardiac crest, as part of the roof of the rhombencephalon, is located between the otic placode and the fourth somite[5,6,7]. A selected cell population migrates through the pharyngeal arches to take residence in a number of organ primordia, including the pharyngeal arch arteries and the heart. On the migration path, neural crest cells (NCC) pass the second heart field and the interaction with the various cell populations results in a complicated differentiation schedule, including the possibility of congenital malformations. NCC are important in differentiation of the wall of the pharyngeal arch arteries, outflow tract separation, valve formation, and differentiation of the conduction system. The contribution by Snider et al.[8] reviews the cell-autonomous polarized movement of the NCC towards the heart and their final destination, entering both the arterial and the venous pole. Using the genetically defined Pax3 (splotch) mutant mouse, presenting, e.g., a persisting truncus arteriosus, the neural crest fate is beautifully illustrated.

A further extracardiac cell population has recently lost its “extracardiac denomination” as it is becoming clear that the proepicardial organ is part of the second heart field and, therefore, only relatively lately added to the primary heart tube, very similar to the outflow tract. In their review, Lie-Venema et al.[9] provide evidence that the proepicardial organ is the base of the epicardium, which transforms through EMT into migrating cells. These epicardium-derived cells (EPDC) invaginate the cardiac wall and provide the large majority of cardiac fibroblasts and coronary smooth muscle cells. Other functions of the EPDC relate to valve formation, the architecture of the cardiac wall, and the differentiation of the Purkinje system.

It is evident that signaling is an important mechanism in embryonic differentiation and the heart, being complex in its development is no exception. Hoover and colleagues[10] concentrate on the role of retinoid signaling in each stage of cardiac development. Retinoid signaling is needed for cardiac lineage determination, tube formation, looping, epicardium formation, ventricular maturation, outflow tract septation, and coronary arteriogenesis. Dietary vitamin A is biologically inert and needs conversion to bioactive retinoic acids, the ligands for the receptors (RXR and RAR). The metabolic pathways and the receptor patterns in time and space dictate the importance in development. Deficiency or surplus of vitamin A[11], and disturbances in the pathways or the receptor function, explain the variety of congenital malformations. The epicardium is particularly interesting as proepicardium and EPDC produce RA, thereby driving ventricular maturation. Endocardial cushions and NCC also show a tight dependency on RA signaling. Interactions of RA with other cardiac regulation pathways, such as Wnt and TGFbeta, are provided.

The development of the cardiac conduction system (CCS) is an elusive phenomenon with many undecided elements, yet to be resolved. Jongbloed et al.[12] provide a thorough review of this complex system used for studying components of the conduction system, including the sinus and atrioventricular nodes, the His and left and right ventricular bundle branches, as well as the Purkinje network. Their study encompasses various so-called markers, such as the T-box and homeodomain transcription factors, transcriptional repressors of the Id family, and the GATA factors. Furthermore, functional characteristics include the (specific) ion channels, such as MinK, the HCN family, and connexins. The intriguing part encompasses the consequences of embryonic (mal)development of the CCS for arrhythmias later in life. It is evident that the left atrial area surrounding the entrance of the pulmonary veins has an embryonic origin that explains this hot spot for arrhythmogenicity. Furthermore, the development of the atrioventricular junction explains the possible persistence of atrioventricular connections, related to Mahaim tachycardia.

Epigenetic factors have been underestimated in their influence on cardiac development. Icardo[13] has been an advocate in this field, while more recently, the influence of mechanical forces of the flowing blood has been described[14,15] as well as the forces emanated by the contractions of the heart tube[16]. Hierck et al.[17] demonstrate in the embryo that biomechanical characteristics of blood flow and accompanying shear sensing by endothelial cells evoke intracellular reactions based temporarily on calcium fluxes and more permanently on gene expression changes, also known in atherosclerotic events[18]. The important mechanosensor is the cytoskeleton that is sensitized by the nonmotile primary cilium extending into the lumen. Differences in high shear stress areas and areas with disturbed or oscillatory flow accompany various expression patterns of shear stress–dependent genes, including nitric oxide synthase-3, krűppel-like factor-2, and endothelin-1.

In conclusion, both intrinsic and extrinsic factors determine the development of the cardiovascular system. These include Wnt and RA signaling; the involvement of extracardiac cell populations, such as neural crest and proepicardiac cells; the origin of the conduction system; and physical aspects, such as hemodynamic forces and material properties, including the role of the cytoskeleton in mechanotransduction.

### REFERENCES

1. Copp, A.J. (1995) Death before birth: clues from gene knockouts and mutations. *Trends Genet.* **11,** 87–93.
2. Conway, S.J., Kruzynska-Frejtag, A., Kneer, P.L., Machnicki, M., and Koushik, S.V. (2003) What cardiovascular defect does my prenatal mouse mutant have, and why? *Genesis* **35,** 1–21.
3. Eisenberg, L.M. and Eisenberg, C.A. (2007) Evaluating the role of Wnt signal transduction in promoting the development of the heart. *TheScientificWorldJOURNAL* **7,** 161–176. [pdf3]
4. Linask, K.K. and VanAuker, M. (2007) A role for the cytoskeleton in heart looping. *TheScientificWorldJOURNAL* **7,** 280–298. [pdf4]
5. Hutson, M.R. and Kirby, M.L. (2007) Model systems for the study of heart development and disease. Cardiac neural crest and conotruncal malformations. *Semin. Cell Dev. Biol.* **18,** 101–110.
6. Boot, M.J, Gittenberger-De Groot, A.C., Van Iperen, L., Hierck, B.P., and Poelmann, R.E. (2003) Spatiotemporally separated cardiac neural crest subpopulations that target the outflow tract septum and pharyngeal arch arteries. *Anat. Rec. A Discov. Mol. Cell Evol. Biol.* **275,** 1009–1018.
7. Verberne, M.E., Gittenberger-de Groot, A.C., van Iperen., L., and Poelmann, R.E. (2000) Distribution of different regions of cardiac neural crest in the extrinsic and the intrinsic cardiac nervous system. *Dev. Dyn.* **217,** 191–204.
8. Snider, P., Olaopa, M., Firulli, A.B., and Conway, S.J. (2007) Cardiovascular development and the colonizing cardiac neural crest lineage. *TheScientificWorldJOURNAL* **7,** 1090–1113. [pdf8]
9. Lie-Venema, H., VandenAkker, N.M., Bax, N.A., Winter, E.M., Maas, S., Kekarainen, T., Hoeben, R.C., deRuiter, M.C., Poelmann, R.E., and Gittenberger-de Groot, A.C. (2007) Origin, fate, and function of epicardium-derived cells (EPDCs) in normal and abnormal cardiac development. *TheScientificWorldJOURNAL* **7,** 1777–1798. [pdf9]
10. Hoover, L.L., Burton, E.G., Brooks, B.A., and Kubalak, S.W. (2007) The expanding role for retinoid acid signaling in heart development. *TheScientificWorldJOURNAL* **8,** 194–211. [pdf10]
11. Broekhuizen, M.L., Bouman, H.G., Mast, F., Mulder, P.G., Gittenberger-De Groot, A.C., and Wladimiroff, J.W. (1995) Hemodynamic changes in HH stage 34 chick embryos after treatment with all-trans-retinoic acid. *Pediatr. Res.* **38,** 342–348.
12. Jongbloed, M.R., Mahtab, E.A., Blom, N.A., Schalij, M.J., and Gittenberger-de Groot A.C. (2008) Development of the cardiac conduction system and the possible relation to predilection sites of arrhythmogenesis. *TheScientificWorldJOURNAL* **8,** 239–269. [pdf12]
13. Icardo, J.M. (1996) Developmental biology of the vertebrate heart. *J. Exp. Zool.* **275,** 144–161.
14. Yoshigi, M., Hu, N., and Keller, B.B. (1996) Dorsal aortic impedance in stage 24 chick embryo following acute changes in circulating blood volume. *Am. J. Physiol.* **270,** H1597–1606.
15. Groenendijk, B.C., Stekelenburg-de Vos, S., Vennemann, P., Wladimiroff, J.W., Nieuwstadt, F.T., Lindken, R., Westerweel, J., Hierck, B.P., Ursem, N.T., and Poelmann, R.E. (2008) The endothelin-1 pathway and the development of cardiovascular defects in the haemodynamically challenged chicken embryo. *J. Vasc. Res.* **45,** 54–68.
16. Taber, L.A. (2006) Biophysical mechanisms of cardiac looping. *Int. J. Dev. Biol.* **50,** 323–332.
17. Hierck, B.P., Van der Heiden, K., Poelma, C., Westerweel, J., and Poelmann, R.E. (2008) Fluid shear stress and inner curvature remodeling of the embryonic heart. Choosing the right lane. *TheScientificWorldJOURNAL* **8,** 212–222. [pdf17]

18. Van der Heiden, K., Hierck, B.P., Krams, R., de Crom, R., Cheng, C., Baiker, M., Pourquie, M.J., Alkemade, F.E., DeRuiter, M.C., Gittenberger-de Groot, A.C., and Poelmann, R.E. (2008) Endothelial primary cilia in areas of disturbed flow are at the base of atherosclerosis. *Atherosclerosis* **196,** 542–550.

**This article should be cited as follows:**

Poelmann, R.E. and Gittenberger-de Groot, A.C. (2008) Cardiac development. *TheScientificWorldJOURNAL:* TSW Development & Embryology **8**, 855–858. DOI 10.1100/tsw.2008.102. [CDed]
